# A novel image encryption framework using Wireworld cellular automaton and hybrid chaotic maps for enhanced security

**DOI:** 10.1371/journal.pone.0332480

**Published:** 2025-11-13

**Authors:** Bayan Alabdullah, Hadeel Alsolai, Fatimah Alhayan, Atif Ikram, Abrar Almjally, Mohammad Shehab, Marwan Ali Albahar

**Affiliations:** 1 Department of Information Systems, College of Computer and Information Sciences, Princess Nourah bint Abdulrahman University, Riyadh, Saudi Arabia; 2 Department of Computer Science & IT, The University of Lahore, Lahore, Pakistan; 3 College of Information Technology, Amman Arab University, Amman, Jordan; 4 Information Management Department, College of Computer and Information Sciences, Imam Mohammad Ibn Saud Islamic University (IMSIU), Riyadh, Kingdom of Saudi Arabia; 5 Department of Computing, College of Engineering and Computing in Al-Lith, Umm Al-Qura University, Makkah, Saudi Arabia; University of Lagos Faculty of Engineering, NIGERIA

## Abstract

The generation, storage, and transmission of digital images have become ubiquitous in today’s interconnected world. Ensuring the security of these images is a critical challenge that demands immediate attention. This study proposes a novel image encryption algorithm designed to address these concerns effectively. The proposed framework leverages the unique properties of three key constructs: the Wireworld cellular automaton, the 1D logistic chaotic map, and the piecewise linear chaotic map. The 1D logistic chaotic map is employed to generate random numbers, which are used to initialize the Wireworld cellular automaton. The automaton, in turn, introduces scrambling effects into the plaintext image, effectively disrupting its pixel arrangement. Additionally, the piecewise linear chaotic map is utilized to achieve diffusion effects, further enhancing the security of the encryption process. Extensive security analyses and machine experiments have yielded highly promising results. The proposed algorithm has been rigorously evaluated using a variety of validation metrics, including key space analysis, correlation coefficient, Cartesian and polar histograms, information entropy, histogram variance, and peak signal-to-noise ratio (PSNR). In particular, we got an entropy of 7.9975 and histogram variance 251.9867. These metrics collectively demonstrate the algorithm’s strong security characteristics and its resilience against potential attacks. The findings suggest that the proposed image cipher is not only highly secure but also practical for real-world applications. It holds significant potential for safeguarding digital images across diverse domains, including healthcare, military, and multimedia communication. This study underscores the viability of the proposed approach as a reliable solution for ensuring the security of images in an increasingly digital world.

## 1 Introduction

There is an urgent need to adapt to the fast-changing world. No matter what field of life we talk about, there is always going to be the element of security attached to it. The massive amount of data that is being produced in everyday life needs to be shared over a network. The element of security needs to be respected during this sharing of information [1–4]. Sometimes people tend to steal this data by disrespecting the privacy of others. Nowadays a lot of this data is in the form of an image. Hence, there is a need to protect the privacy of people and design a system which is secure to all sorts of threats. Over the course of our history, some major works have been done to protect textual data. To name a few, we have Data Encryption Algorithm (DES), Advanced Encryption Algorithm (AES), and RSA [5]. It has become a necessity to protect data that is in the form of an image.

Chaotic systems have been widely utilized to generate streams of random numbers for cryptographic applications. These systems can be categorized into low-dimensional, high-dimensional, and hyperchaotic systems. Low-dimensional systems typically produce one or two random sequences, while higher-dimensional and hyperchaotic systems are capable of generating multiple streams simultaneously.

A variety of one-dimensional (1D) chaotic maps—such as the sine map, logistic map, tent map, and cubic map—have been studied and applied in image encryption algorithms [6]. These maps usually yield a single stream of pseudo-random numbers, which are primarily employed to perform confusion and diffusion operations on the pixel values of an image.

In addition to 1D maps, two-dimensional (2D) chaotic maps are also commonly used. As the name suggests, 2D maps produce two streams of random numbers, making them suitable for more complex encryption schemes. Notable works such as [7–9] have successfully integrated various 2D chaotic maps into image encryption frameworks.

Moreover, a wide range of higher-dimensional chaotic systems have been proposed to generate more than two streams of pseudo-random numbers. Examples include the Intertwining Logistic Map [10], 4D chaotic maps [11], and 5D chaotic maps [12], all of which have demonstrated promising results in enhancing the security and complexity of image encryption schemes.

In the current years, cryptographers have paid an increasing attention to the security of digital images. For this purpose, novel encryption and decryption algorithms have been developed. For instance, the work [13] wrote a novel image encryption algorithm which consists of some phases. First, the least significant bit (LSB) of a randomly selected pixel from the plaintext image was modified. The hash value of the modified image was then extracted and used as both the initial condition and control parameter for the proposed hyper-chaotic system, enhancing resistance against plaintext-based attacks. Next, pixel confusion within the encrypted image was strengthened through bit-plane shifting and a novel crossover-box mechanism for pixel swapping. Finally, to further improve security and computational efficiency, a combination of forward and backward diffusion (FDBD) was applied, augmented with the integration of perturbation factors. Apart from that, the study [14] employed non-chain ring theory for image encryption by establishing a bijective mapping between the group of units and the Galois field. This theoretical foundation enabled the construction of a novel substitution box (S-box) that leverages the inherent algebraic structure of the unit group. The encryption process involved a sequence of operations, including pixel transformation, partitioning, field inversion, and exclusive OR (XOR) computations. To assess the cryptographic strength of the proposed method, the authors conducted a comprehensive evaluation, encompassing S-box performance metrics, statistical analysis, differential analysis, computational complexity, and NIST randomness tests. The results demonstrated that the encryption scheme was robust against a wide range of established cryptographic attacks and is capable of producing ciphertext with strong randomness and unpredictability characteristics. Apart from that, these works [15–17] rendered novel algorithms of the image encryption.

Cryptanalysis serves as a parallel and essential discipline that investigates the vulnerabilities, weaknesses, and structural flaws in image encryption algorithms. Several studies have successfully broken existing image ciphers, as demonstrated in [18–21]. These findings highlight the ongoing need for the development of more secure and resilient image encryption schemes.

When it comes to encrypting an image, scrambling/confusion and diffusion are approached differently by different researchers [22–32]. A research [33] based on the combination of logistic map, magic square, (DCT) discrete cosine transform and Schur decomposition provided us with a cipher that can be used to encrypt stereo images. The algorithm started with the unification of images with logistic map and DCT. Later, the application of Schur decomposition and the method of magic square rendered the cipher image as an output. In an other study [34], three levels for an image encryption algorithm were proposed. The first level comprised of 7D hyperchaotic map. The second level applied an S-box of Extended Cellular Automata (CA). Lastly, the third level utilized rule 30 of CA. Security analysis and the machine simulations rendered very competitive results.

A yet another research work [35] utilized machine learning techniques namely, genetic algorithm and neural networks along with mathematical construct Latin square to come up with a novel image encryption algorithm. In this particular work, random sequence was generated using neural networks. The final cipher image was obtained by carrying out an XOR operation between the Latin square and the input matrix. This was done for a finite time to make an encrypted image population. Column and row arrangements from the randomly selected two parents resulted in the offspring. In order to obtain the better cipher image, genetic algorithm was used in such a way so that the pixel correlation value may be minimized. Vast security analysis and the machine experimentation indicated that the cipher was very robust and defiant to the varied attacks launched by the hackers.

Focusing on a big parametrized interval, a technique [36] based on one-dimensional sine chaotic system (1DSCS) was introduced. Scrambling was performed in two parts. Firstly, column and row were utilized to boost the security effects. Secondly, Arnold Map was ignited to spawn the streams of random numbers. After scrambling, dynamic diffusion was performed by four formulas. The formula selection was made according to the values of random numbers given by 1DSCS. The algorithm proved to be very resistant to common cipher attacks.

An image encryption algorithm based on filtration of images, sequence DNA operations and memrisitve chaotic system was given in [37]. Moreover, preprocessing of the input image was made by dynamic image filtering (STDIF). In the next step, DNA sequence encoding rules were decided, which were generated from the chaotic systems and the information taken from input image. Apart from that, the permutation operation was performed which displaced each element randomly with the usage of DR3DMS (double random 3D matrix scrambling). On top of this, resistance against attacks was increased by performing plane diffusion with the use of 3D DNA matrix. Lastly cipher image was obtained using the decoding rules as suggested by the DNA sequence. Additionally, an SHA-256 hash function was chosen to select the different key stream values.

In an other study [38], an algorithm utilizing multiple image encryption and Zigzag transformation was proposed. This algorithm was designed to prevent stealing of images over a network transmission. Initially, the input plaintext images were represented as a cube. Moreover, Henon map was introduced for the selection of first point in the 2D Zigzag transformation. Apart from that, image scrambling was done by stereo Zigzag transformation. In the last step, diffusion operation was taken place by a chaotic sequence. All these steps rendered the required cipher image.

The combination of the Logistic map and Sine map resulted in an enhanced version of 2D chaotic maps [39]. Additionally, the reported study introduced a novel 4D chaotic map. The proposed algorithm integrated DNA coding, an improved 2D chaotic map, and a 4D chaotic system. The pixel correlation from the three RGB color channels was analyzed and arranged in ascending order. By iterating the 4D chaotic system, a chaotic sequence was generated, which was then utilized in the encryption process through DNA encoding.

Many image ciphers are replete with too many loopholes and other lacunas in the core of their design strategies. For example, the paper [40] presented an analysis of the Image Encryption Algorithm which employed the notions of Cellular Automata and the Chaotic Skew Tent Map (IEA-CACSTM) [41]. Various numerical analyses were carried out to judge the security of IEA-CACSTM. While the algorithm [41] claimed to be defiant and secure to various attacks, including well known chosen-plaintext attacks, the cryptanalysis revealed significant vulnerabilities. Specifically, the three random numbers and two index matrices generated by IEA-CACSTM were independent of the plaintext, leading to the potential existence of equivalent permutation keys. In the case of a chosen-plaintext attack, there was a high possibility to deduce the answer of modulus operation associated with one of the chaotic numbers, as well as certain initial conditions. Based on this, a row-by-row decryption technique was proposed to recover the permuted image, ultimately enabling the decryption of plaintext images of varied dimensions using equivalent permutation keys. Lastly, numerical simulations and theoretical analysis validated the effectiveness of the decryption approach. Moreover, these works [21,42–44] explain the cryptanalysis of the published image ciphers.

Inspired by the above discussion, the current research work crafts a yet another image cipher (Wireworld Cellular Automaton and Chaotic Maps-Based Image Cipher (WCA-CMC)) using the constructs of Wireworld cellular automaton, 1D logistic chaotic map and the piecewise linear chaotic map. The chaotic maps facilitate in generating the streams of random numbers. Whereas, the mathematical construct of Wireworld cellular automaton helped in realizing both the confusion and diffusion operations necessary for developing the required security product.

### 1.1 Research hypothesis

The central hypothesis of this research is that the integration of Wireworld cellular automaton with one-dimensional logistic and piecewise linear chaotic maps can significantly enhance the security of image encryption systems by providing stronger confusion and diffusion capabilities. This hybrid approach is expected to generate a cipher with high randomness, resistance to common cryptanalytic attacks, and superior statistical performance compared to traditional methods. Apart from that, following bullets characterize the current research endeavor.

- A novel image encryption algorithm, WCA-CMC, is proposed by integrating Wireworld Cellular Automaton with 1D logistic and piecewise linear chaotic maps to harness both structural dynamics and randomness for secure image scrambling.
- The chaotic maps are employed to generate key-dependent pseudo-random sequences, while the Wireworld CA facilitates the realization of confusion and diffusion through its evolving cell interactions.
- The proposed cipher demonstrates high entropy, low correlation, and strong resistance against statistical and differential attacks, validating its robustness over conventional encryption approaches.

The remainder of this article is structured as follows: Sect 2 provides an overview of chaotic systems and the Wireworld cellular automaton utilized in the core of the proposed algorithm. Sect 3 details the generation of random data and the development of the proposed image encryption scheme. Sect 4 presents experimental results based on four grayscale images. In Sect 5, security analysis and performance evaluation of the algorithm are conducted. A Discussion Sect 6 defends the hypothesis made in the Introduction section. Finally, Sect 7 concludes the paper with key findings, concluding remarks, and potential directions for future research.

## 2 Building blocks

### 2.1 Overview of cellular automata with emphasis on the Wireworld model

Cellular automata (CAs) are dynamical systems that exhibit complex global behavior emerging from simple local interactions and computations. Since their inception by John von Neumann in the 1950s, CAs have attracted significant attention from researchers across diverse disciplines for modeling a wide range of physical, natural, and real-life phenomena. Traditionally, CAs are uniform; however, non-uniformity has also been explored in aspects such as update patterns, lattice structures, neighborhood dependencies, and local rules [45].

CAs have been extensively used by the image cryptographers to carry out the permutation operations over the pixels of the given images. Table 1 shows some chosen automatons.

**Table 1:** Overview of cellular automata.

Automaton	Description
Elementary Cellular Automata	In mathematics and computability theory, an elementary cellular automaton is a one-dimensional system in which each cell can be in one of two states (typically labeled 0 and 1). The state of a cell in the next generation is determined solely by its current state and the states of its two immediate neighbors. Notably, Rule 110 is an elementary cellular automaton capable of universal computation, making it one of the simplest known models of computation [47].
Quantum Cellular Automata	A quantum cellular automaton (QCA) is an abstract model of quantum computation, inspired by the conventional cellular automata framework introduced by John von Neumann. QCAs have garnered significant attention due to their extremely small feature size—potentially at the molecular or even atomic scale—and their ultra-low power consumption, making them a promising candidate for future computation systems. [48].
Von Neumann cellular automaton	The Von Neumann cellular automaton is one of the earliest and most influential models of cellular automata, introduced by John von Neumann in the 1940s to study self-replication and complex systems. It operates on a two-dimensional orthogonal grid where each cell has five neighbors—the central cell plus its four orthogonal neighbors (north, south, east, west). The model was originally developed to demonstrate the possibility of self-replicating machines, making it foundational in the field of artificial life [49,50].
Moore neighborhood	The Moore neighborhood in cellular automata refers to a two-dimensional square lattice consisting of a central cell and its eight surrounding cells, analogous to 8-connected pixels in computer graphics. Unlike the von Neumann neighborhood, it includes diagonal neighbors. A well-known example utilizing this neighborhood is Conway’s Game of Life [51,52].
Totalistic Cellular Automaton	A totalistic cellular automaton determines the future state of a cell based only on the total or average value of the cells in its neighborhood, ignoring the specific arrangement of those values. In the one-dimensional case, this typically involves the sum or average of the cell itself, its left neighbor, and its right neighbor. The evolution rules can be fully described in a table mapping these totals to the cell’s next state [53].
Wireworld Cellular Automaton	Wireworld is a two-dimensional cellular automaton introduced by Silverman in 1987, later popularized by Dewdney. It simulates digital electronic circuits using simple rules and four cell states: empty, electron head, electron tail, and conductor. The next state of each cell depends on its current state and the states of the eight surrounding cells in the Moore neighborhood. Despite its simplicity, Wireworld is Turing complete and capable of modeling complex circuit behavior [54].

According to the Table 2, the entropy values for both Arnold’s cat map and Wireworld CA are identical, indicating similar overall randomness levels. However, the correlation coefficients reveal significant differences: Wireworld CA effectively decorrelates pixel values in all directions, with values close to zero, while Arnold’s Cat Map retains high positive correlations, suggesting weaker scrambling capability.

**Table 2:** Comparison of Wireworld CA and Arnold’s Cat Map in terms of entropy and correlation coefficients.

Method	Entropy	Horizontal Corr.	Vertical Corr.	Diagonal Corr.
Wireworld CA	7.5954	-0.0586	-0.0825	0.1051
Arnold’s Cat Map	7.5954	0.1182	0.1191	0.1131

Wireworld is a two-dimensional cellular automaton that simulates the behavior of electrons flowing through wires, making it a versatile and computationally efficient model for various applications, including image encryption [46]. Unlike traditional CA models, Wireworld operates on a mesh of cells, each of which can exist in one of four states: *empty*, *wire*, *electron head*, or *electron tail*. The state transitions are governed by a set of simple yet powerful rules:

- An *electron head* becomes an *electron tail* in the next iteration.
- An *electron tail* becomes a *wire* in the next iteration.
- A *wire* becomes an *electron head* if it is adjacent to exactly one or two *electron heads*.
- An *empty* cell remains *empty* in all iterations.

These rules enable Wireworld to simulate complex signal propagation and logic operations, such as the creation of logic gates, oscillators, and even computational circuits. The automaton’s ability to generate chaotic and unpredictable patterns from simple initial configurations makes it particularly suitable for cryptographic applications.

**Fig 1:**
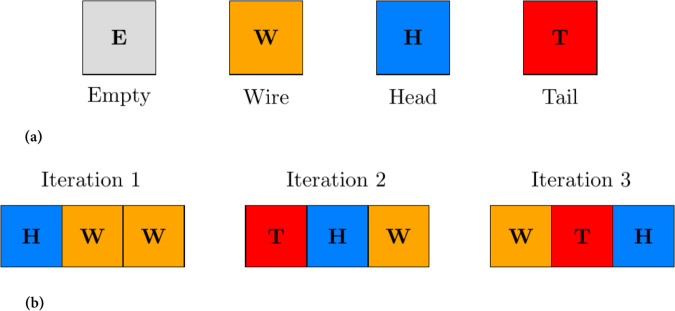
Wireworld and its evolution. (a) Four states of Wireworld; (b) Evolution of four states.

The four states of Wireworld are visually represented in Fig 1a. Fig 1b demonstrates how Wireworld evolves over iterations.

- In **Iteration 1**, the electron head (**H**) is at the first cell, followed by two wires (**W**).
- In **Iteration 2**, the head becomes a tail (**T**), and the adjacent wire becomes a new head (**H**).
- In **Iteration 3**, the tail becomes a wire (**W**), and the process continues, simulating the flow of electrons.

This simple yet powerful behavior of Wireworld makes it suitable for cryptographic applications, as it can generate complex and unpredictable patterns from simple initial configurations. By mapping image pixels to Wireworld cells and iteratively applying the automaton’s rules, the pixel values are scrambled in a highly non-linear and secure manner. This process ensures that even a small change in the input image or encryption key results in a significantly different encrypted output, thereby enhancing security. The lightweight nature of Wireworld also makes it an attractive option for resource-constrained environments, such as IoT devices or mobile applications. The following sections delve into the design and implementation of a Wireworld-based image encryption framework, highlighting its security and performance advantages.

### 2.2 Chaotic systems/maps.

#### 2.2.1 Logistic chaotic map

The 1D logistic map is a classic and widely studied example in chaos theory, showcasing how complex and chaotic behavior can arise from a simple deterministic system. It is defined by the following recurrence relation [55]:

$$x_{n+1}=r\cdot x_{n}\cdot (1-x_{n})$$ (1)

Where n=0,1,2,..... and 0<*x*_0_<1. In this equation, *x*_*n*_ denotes the system’s state at the *n^th^* iteration, while *r* serves as a control parameter that influences the system’s behavior. The logistic map is widely recognized for its diverse dynamical properties, ranging from stable fixed points to chaotic oscillations, depending on the value of *r*.

For various values of parameter *r*, logistic map can exhibit the following behaviors [56]:

- *Fixed Points:* For specific values of *r*, the system reaches a stable fixed point. This behavior is observed when *r* falls within certain ranges, particularly when 1 < *r* < 3.
- *Periodic Orbits:* As *r* increases, the system may display periodic behavior, causing its state to oscillate between a specific set of values. This periodic behavior occurs within the range 3<*r*<3.57, where the period undergoes successive doublings through a series of bifurcations.
- *Chaos:* When *r* exceeds approximately 3.57, the system enters a chaotic regime. In this phase, the system demonstrates extreme sensitivity to initial conditions, leading to a seemingly random and highly complex evolution of its state over time.
- *Period-Doubling Bifurcation:* As *r* continues to increase, the logistic map experiences a sequence of bifurcations, with the oscillation period doubling at each step until the system ultimately transitions into chaos.

The logistic map holds significant importance in chaos theory as it offers a straightforward yet powerful model for examining the transition from ordered to chaotic behavior. It stands as a fundamental example in the study of dynamical systems and chaotic phenomena. The map’s hallmark feature is its extreme sensitivity to initial conditions, commonly known as the *butterfly effect*, which illustrates how even tiny changes can lead to vastly divergent outcomes, a characteristic trait of chaotic systems.

**Fig 2:**
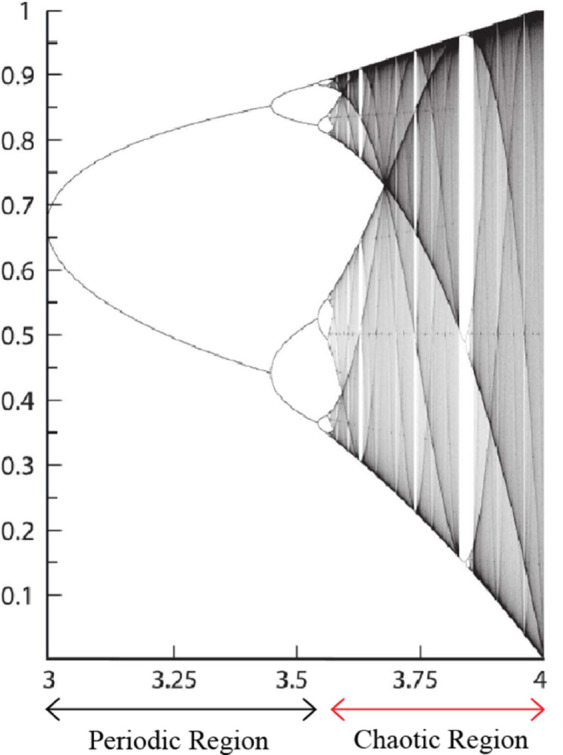
Bifurcation diagram of logistic map.

To gain deeper insights into the logistic map’s behavior, it is often helpful to visualize its attractors and analyze the bifurcation diagram (Fig 2). This diagram illustrates how the system’s long-term behavior evolves as the parameter *r* is varied. The bifurcation diagram unveils the intricate structure of the map’s dynamics, showcasing the transitions between different behavioral regimes, such as fixed points, periodic cycles, and chaos.

**Fig 3:**
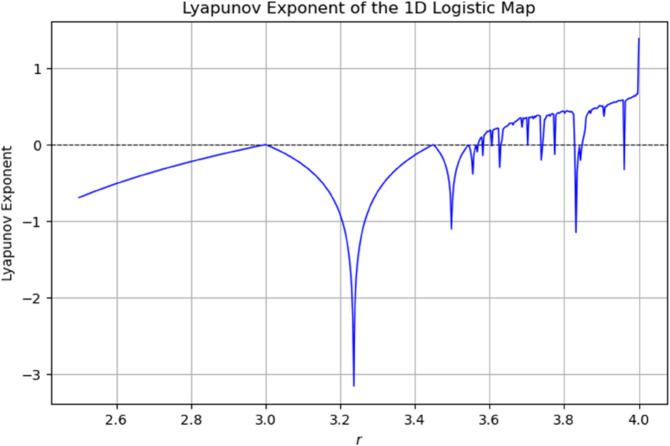
Lyapunov exponent of logistic map.

The Lyapunov exponent diagram of the 1D logistic map, shown in Fig 3, illustrates the system’s transition from periodic to chaotic behavior as the control parameter *r* increases. Negative exponent values indicate stable, periodic dynamics, whereas positive values signify sensitive dependence on initial conditions—a hallmark of chaos. The exponent crosses zero near the onset of chaos, with the largest positive values observed in the fully developed chaotic regime ( r≈4), confirming the map’s strong unpredictability in this range.

#### 2.2.2 Piece-wise linear chaotic map

Among many chaotic maps/systems, there is a Piecewise Linear Chaotic Map (PWLCM) being a one-dimensional mathematical model, defined by the Equation 2 [57]. As evident from the equation, this map exhibits a recursive nature, as it references itself in its formulation.

$$q_{i}=R(q_{i-1},\eta )=\left\{ \begin{array}{cc} \frac{q_{i-1}}{\eta }, & \text{if}\;0<q_{i-1}<\eta \\ \frac{q_{i-1}-\eta }{2-\eta }, & \text{if}\;\eta \leq q_{i-1}<0.5\\ R(1-q_{i-1},\eta ), & \text{if}\;0.5\leq q_{i-1}<1 \end{array}\right.$$ (2)

**Fig 4:**
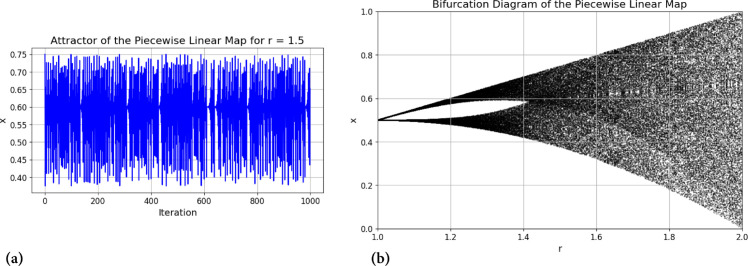
Attractor and bifurcation diagram of the piecewise linear chaotic map. (a) Attractor of piecewise linear chaotic map for *r* = 1.5; (b) Bifurcation diagram of piecewise linear chaotic map.

Where i=0,1,2,...... and 0<*q*_0_<1. For values of *η* within the range (0,0.5), the map exhibits highly desirable chaotic behavior. Additionally, this map is characterized by an even distribution and excellent ergodic properties. Fig 4 displays the attractors and bifurcation diagram of the piecewise linear chaotic map, illustrating its dynamic behavior and structural complexity.

**Fig 5:**
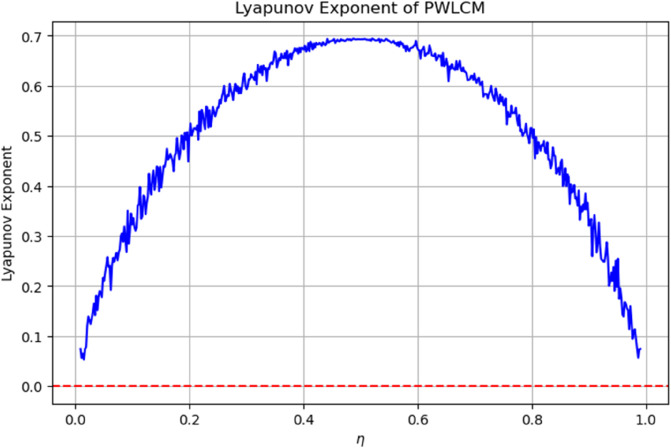
Lyapunov exponent of PWLCM.

The Lyapunov exponent of the Piecewise Linear Chaotic Map (PWLCM) was calculated to evaluate its chaotic behavior across different control parameter values *η*. As shown in Fig 5, the exponent remains positive for almost the entire range of *η*, indicating strong sensitivity to initial conditions and sustained chaotic dynamics. This property ensures that even a minute variation in the initial state leads to significant divergence over time, which is highly desirable for cryptographic applications. The presence of positive exponents confirms that PWLCM can provide robust confusion and diffusion in the proposed image encryption scheme.

The choice of the 1D Logistic map and the PWLCM in the proposed encryption scheme is motivated by their simplicity, ease of implementation, and proven cryptographic strength. Both maps exhibit strong chaotic properties, including large positive Lyapunov exponents, uniform invariant distributions, and high sensitivity to initial conditions. While 2D and hyperchaotic maps can offer a higher degree of complexity, they also demand significantly greater computational resources, which may reduce encryption speed and increase hardware implementation costs. The selected 1D maps strike an optimal balance between security and efficiency, making them suitable for real-time and resource-constrained encryption scenarios, while still delivering high key sensitivity, resistance to common attacks, and excellent statistical performance.

## 3 Wireworld cellular automaton and chaotic maps-based image cipher (WCA-CMC)

**Fig 6:**
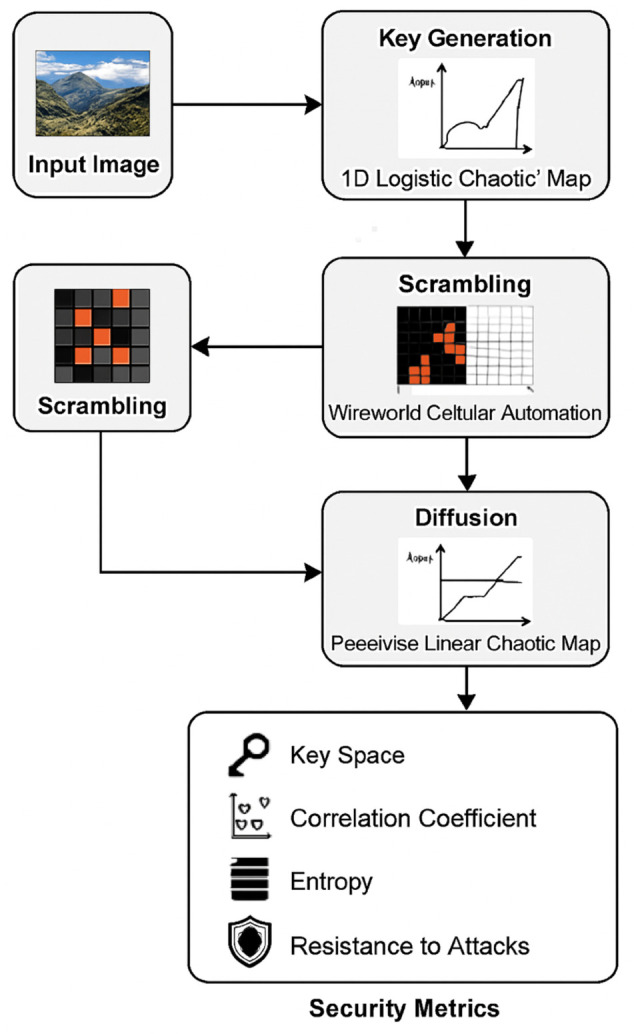
Proposed methodology.

The proposed methodology has been shown in the Fig 6. In the following steps, we will explain the proposed algorithm.

**Step 1:** Plaintext sensitivity is a very important factor while developing any cryptographic product. Its incorporation in any design principle facilitates in defying the potential differential attacks on the cipher. We have taken the average value of the given input plain image *image* and used the following equation to update the initial key *x*_0_ of Chaotic System (1) currently in use.

$$x_{0}^{\prime }=x_{0}+\frac{avg}{2^{50}}$$ (3)

**Step 2:** Call the Algorithm 1 with the list of parameters *image*, $x_{0}^{\prime }$, *r*, *ϕ*, *η*. Line 1 extracts the dimensions of the given image *image* and saves it in (*rows*,*cols*).

**Algorithm 1. WCA-CMC.**

**Input:** *image, *x**_*0*_*, *r**_*0*_*, *ϕ*, *η**

**Output:** $image^{\prime }$

1: rows,cols=image.shape

2: $initial\_state=PopulateInitialState(rows,cols,x_{0},r)$

3: scrambling_pattern=GenerateScramblingPattern(image.shape,ϕ,initial_state)

4: flattened_image=image.flatten()

5: scrambled_flat=np.zeros_like(flattened_image)

6: flat_pattern=scrambling_pattern.flatten()

7: **for** *i* in range(rows): **do**

8:   **for** *j* in range(columns): **do**

9:    idx=i×cols+j

10:    new_pos=flat_pattern[idx]

11:    scrambled_flat[new_pos]=flattened_image[idx]

12:   **end for**

13: **end for**

14: scrambled_image=scrambled_flat.reshape((rows,cols))

15: Ignite the Chaotic map *R* (Equation 2 ) with the seed value *q*_0_ and system parameter *η* which renders the mask image *mask*.

$$\{q_{k}=R(q_{k-1},\eta )\;mask_{k}=(q_{k}\times 10^{14})\;\mathrm{mod}\;256$$ (4)

where 1≤k≤rows×cols.

16: cipher_image=CircularShiftImage(scrambled_image,mask)

17: return cipher_image

**Step 3:** Line 2 calls the Algorithm 2 with the list of parameters *rows*,*cols*,*x*_0_,*r* to initialize the initial state initial_state of the automaton.

**Step 4:** Line 1 of Algorithm 2 calculates the total number of cells in the automaton and assigns it the variable total_cells. Line 2 calls the Algorithm 3 using the arguments $x_{0},r,total\_cells$.

**Algorithm 2. PopulateInitialState.**

**Input:** *rows*, *cols*, *x*_0_, *r*

**Output:** initial_state

1: total_cells=rows×cols

2: $chaotic\_sequence=LogisticMap(x_{0},r,total\_cells)$

3: initial_state=(chaotic_sequence×4).astype(int)

4: initial_state=initial_state.reshape((rows,cols))

5: return initial_state

**Step 5:** Line 1 of Algorithm 3 uses the builtin function *zeros* of the NumPy object *np* with argument *n*. This function creates a NumPy array of size *n* consisting of zeros and assigns it to the variable *sequence*. Line 2 assigns the seed value *x*_0_ to *sequence*[0].

**Algorithm 3. LogisticMap.**

**Input:** *x*_0_, *r*, *n*

**Output:** *sequence*

1: sequence=np.zeros(n)

2: *sequence[*0*]* = *x*_0_

3: **for** *i* in range(1,n+1) **do**

4:   sequence[i]=r×sequence[i−1]×(1−sequence[i−1])

5: **end for**

6: return *sequence*

**Step 6:** Lines (3-4) populate the array *sequence* with random numbers using the Chaotic Map (1). Line 6 returns this array to the calling Algorithm 2 which assigns it to the array chaotic_sequence. Line 3 normalizes the random numbers to the range [0, 3]. *astype*(*int*) is a builtin function of the NumPy arrays which truncates the decimal part. Lines (4-5) reshape the initial_state array to (*rows*,*cols*) and return it to Algorithm 1 which assigns this array again to the array variable initial_state.

**Step 7:** Call the Algorithm *GenerateScramblingPattern* with the parameters image.shape,ϕ,initial_state. The mandate of this algorithm is to scramble and shuffle the 2D array initial_state. *ϕ* is a part of the secret key which controls the degree of randomness.

**Step 8:** Line 2 of Algorithm 4 assigns the 2D array initial_state to an other 2D array of wireworld_mesh. *for* loop at line 3 iterates for *ϕ* times. In each iteration, the Algorithm 5 *WireworldStep* is being invoked with the parameter wireworld_mesh. The Algorithm 5 copies the 2D array wireworld_mesh to the variable *mesh* which becomes an other 2D array.

**Step 9:** The nested *for* loops at the lines 3 and 4 sweep through the entire *mesh* corresponding to the Wireworld cellular automaton. The cells of this automaton are updated depending on the *if* conditions at lines 5, 7, 9 and 21. Lastly, line 31 returns the updated automaton named as new_mesh to the Algorithm 4 at the line 4, which in turn, assigns this 2D array to an other 2D array $wireworld\_mesh^{\prime }$. Line 5 assigns this array $wireworld\_mesh^{\prime }$ to the previous array wireworld_mesh for the next iteration. This process recurs for *ϕ* times to boost the security effects.

**Step 10:** The line 7 (Algorithm 4) first flattens the 2D mesh wireworld_mesh into a 1D array, then applies *np*.*argsort*() to return the indices that would sort this array in ascending order. Finally, it reshapes the sorted indices back to the original mesh dimensions (rows×cols), preserving the spatial structure of the sorted data. Moreover, the resultant 2D array is being assigned to a yet another 2D array of scrambling_pattern. Finally, line 8 returns this array to the Algorithm 1 at line 3.

**Algorithm 4. GenerateScramblingPattern.**

**Input:** *shape, *ϕ*, initial_state*

**Output:** scrambling_pattern

1: rows,cols=shape

2: wireworld_mesh=initial_state

3: **for** _ in range(ϕ): **do**

4:   $wireworld\_mesh^{\prime }=WireworldStep(wireworld\_mesh)$

5:   $wireworld\_mesh=wireworld\_mesh^{\prime }$

6: **end for**

7: scrambling_pattern=np.argsort(wireworld_mesh.flatten()).reshape(rows,cols)

8: return scrambling_pattern

**Step 11:** Lines (4 - 6) flatten the 2D image *image* and the scrambling pattern scrambling_pattern into 1D arrays. Then, for each pixel at position (*i*,*j*), its linear index *idx* is calculated (Lines 7 - 9). The pixel is then relocated to a new position new_pos in the scrambled image based on the corresponding value in the flattened scrambling pattern (Lines 10 - 11). This reordering of pixels enhances the image’s security by disrupting its original structure. Lastly, line 14 reshapes the scrambled_flat image to the dimensions of (*rows*,*cols*).

**Step 12:** By providing the seed value *q*_0_ and the system parameter *η*, the chaotic map *R* of Equation (2) produces the mask image *mask* having values in the range of [0, 255]. This will be used to embed the diffusion effects in the scrambled image scrambled_image.

**Algorithm 5. WireworldStep.**

**Input:** *mesh*

**Output:** new_mesh

1: rows,cols=mesh.shape

2: new_mesh=np.zeros_like(mesh)

3: **for** *i* in range(rows): **do**

4:   **for** *j* in range(columns): **do**

5:    **if** mesh[i,j]==2: **then**

6:     new_mesh[i,j]=3

7:    **else if** mesh[i,j]==3: **then**

8:     new_mesh[i,j]=1

9:    **else if** mesh[i,j]==1: **then**

10:     *neighbors* = [

11:     *mesh*[(*i*–1) % *rows*,(*j*–1) % *cols*],

12:     *mesh*[(*i*–1) % *rows*,*j*],

13:     *mesh*[(*i*–1) % rows,(j+1) % *cols*],

14:     *mesh*[*i*,(*j*–1) % *cols*],

15:     mesh[i,(j+1) % *cols*],

16:     mesh[(i+1) % *rows*,(*j*–1) % *cols*],

17:     mesh[(i+1) % *rows*,*j*],

18:     mesh[(i+1) % rows,(j+1) % *cols*],

19:     ]

20:     electron_head_count=neighbors.count(2)

21:     **if** electron_head_count==1 or electron_head_count==2: **then**

22:      new_mesh[i,j]=2

23:     **else**

24:      new_mesh[i,j]=1

25:     **end if**

26:    **else**

27:     new_mesh[i,j]=0

28:    **end if**

29:   **end for**

30: **end for**

31: return new_mesh

**Step 13:** Line 16 calls the Algorithm 6 with the parameters scrambled_image and *mask*. This algorithm performs a pixel-wise shifting operation on a scrambled image scrambled_image using a corresponding mask image *mask*. For each pixel (*i*,*j*) (*for* loop at lines 4-5), the shift amount is determined by *mask*[*i*,*j*] % max_shift (Line 7), where max_shift is set to 8 (Line 2). If the mask value is even (Line 8), the pixel undergoes a right circular shift followed by a left shift to maintain 8-bit integrity (Line 9). If the mask value is odd (*else* statement on line 10), the shifts are reversed (left first, then right) (Line 11). The bitwise AND operation & ensures that the result stays within the 8-bit range. This operation enhances diffusion in image encryption by introducing non-linear transformations. Finally, line 16 returns the diffused image shifted_image to the Algorithm 1 at line 16. Line 17 returns the final cipher image cipher_image.

**Algorithm 6. CircularShiftImage.**

**Input:** scrambled_image*, mask*

**Output:** shifted_image

1: shifted_image=np.zeros_like(scrambled_image)

2: max_shift=8

3: rows,cols=scrambled_image.shape

4: **for** *i* in range(rows): **do**

5:   **for** *j* in range(columns): **do**

6:    pixel=scrambled_image[i,j]

7:    shift=mask[i,j] % max_shift

8:    **if** *mask*[*i*,*j*] % 2==0: **then**

9:     shifted_pixel=((pixel>>shift)|(pixel<<(max_shift−shift)))

& ((1<<max_shift)−1)

10:    **else**

11:     shifted_pixel=((pixel<<shift)|(pixel>>(max_shift−shift)))

& ((1<<max_shift)−1)

12:    **end if**

13:    shifted_image[i,j]=shifted_pixel

14:   **end for**

15: **end for**

16: return shifted_image

The current research project has been done using the principles of private key cryptography. So, the decryption algorithm will be just a reversal of the steps of the encryption algorithm.

## 4 Experimentation and simulation

In this section, we have selected the four grayscale images named as Hailstones, Flowers, Chair and the Bride for the sake of simulation and security analysis. All these images have the size of 256×256. Moreover, the machine experimentation was conducted using the Python 3 tool.

**Fig 7:**
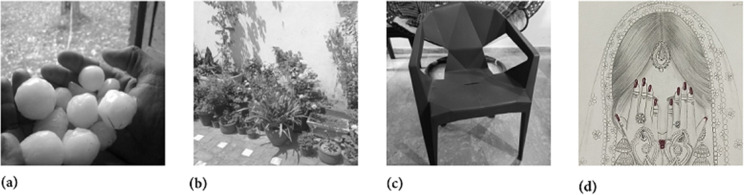
Original test images: (a) Hailstones image; (b) Flowers image; (c) Chair image; (d) Bride image.

**Fig 8:**
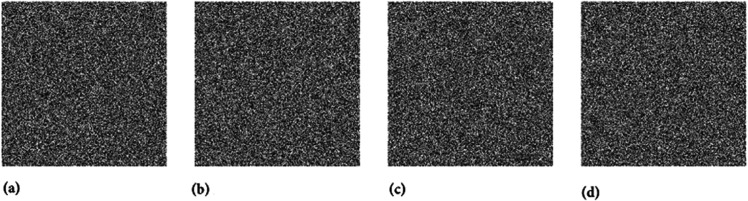
Images after encryption: (a) Hailstones image; (b) Flowers image; (c) Chair image; (d) Bride image.

**Fig 9:**
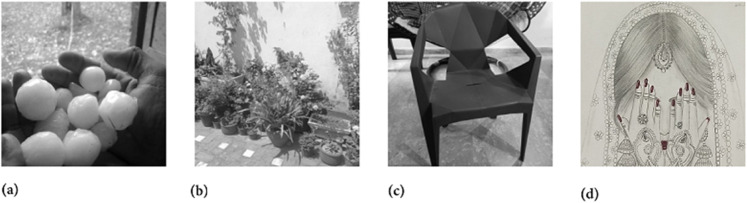
Images after decryption: (a) Hailstones image; (b) Flowers image; (c) Chair image; (d) Bride image.

**Fig 10:**
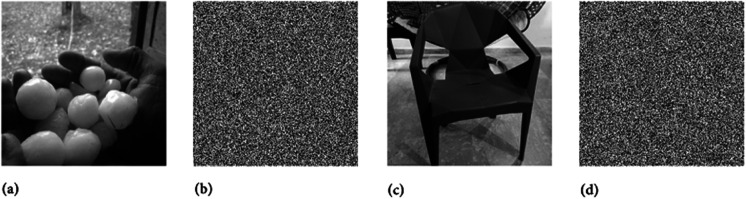
Encryption and decryption of 512×512 images: (a) Hailstones plain image; (b) Hailstones cipher image; (c) Chair plain image; (d) Chair cipher image.

Two chaotic maps have been employed in this study. First is the 1D logistic chaotic map which carried out the scrambling project of the proposed image cipher. Its initial value and the system parameters are: *x*_0_ = 0.5 and *r* = 0.3. Apart from that, the second chaotic map is PWLCM which facilitated in realizing the diffusion effects in the cipher. Its initial value and the system parameter are *q*_0_ = 0.4 and η=0.2. Additionally, *ϕ* has been set as $\phi =2^{32}$. Figs 7, 8, and 9 respectively illustrate the plaintext input images, the encrypted images, and the retrieved images. It is evident that the plaintext images have been completely transformed into indistinct, cloud-like forms, leaving no discernible trace of the original content. This demonstrates the effectiveness of the encryption process and its successful implementation. Furthermore, the cipher images have been accurately reconstructed into their original forms, reaffirming the robustness of the proposed decryption mechanism. Addtionally, Fig 10 shows the application of encryption and decryption machineries on the 512×512 sized images.

**Fig 11:**
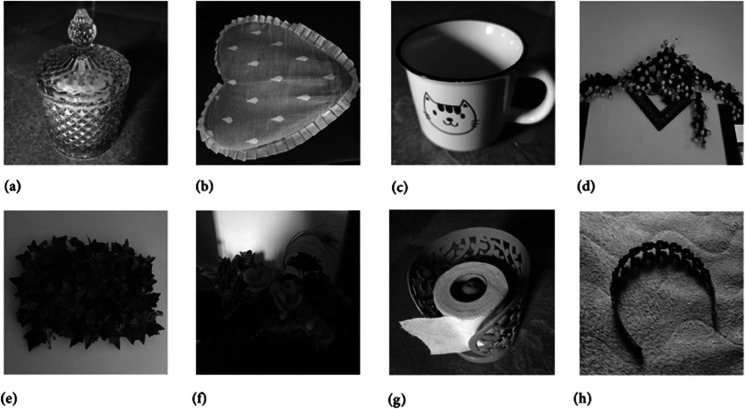
Original test images: (a) Sugar jar image; (b) Cushion image; (c) Cup image; (d) Decoration piece image (e) Leaves image; (f) Flowers image; (g) Tissue roll image; (h) Hair catch image.

**Fig 12:**
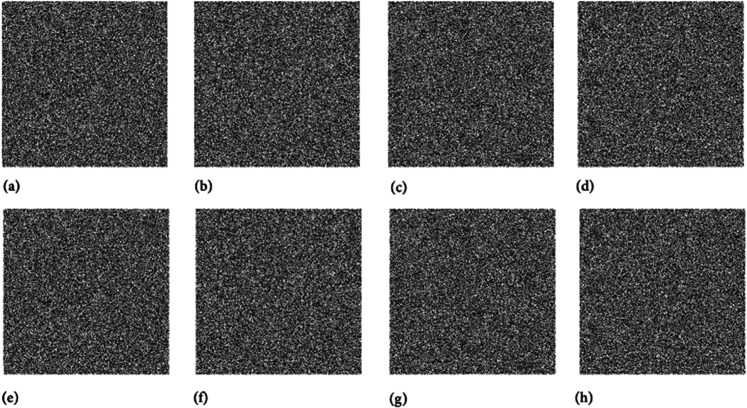
Encrypted images: (a) Sugar jar image; (b) Cushion image; (c) Cup image; (d) Decoration piece image (e) Leaves image; (f) Flowers image; (g) Tissue roll image; (h) Hair catch image.

**Fig 13:**
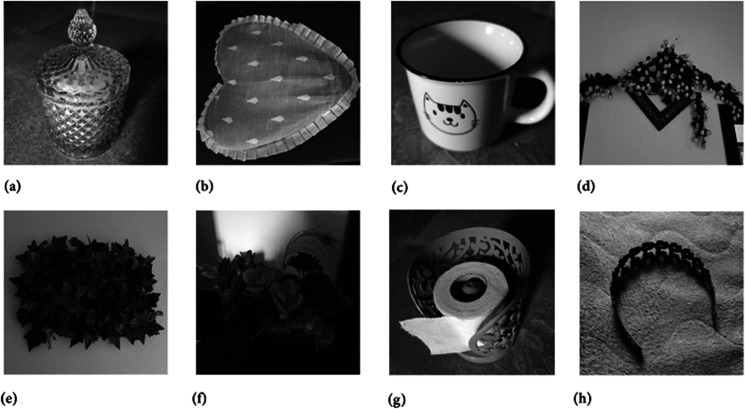
Decrypted images: (a) Sugar jar image; (b) Cushion image; (c) Cup image; (d) Decoration piece image (e) Leaves image; (f) Flowers image; (g) Tissue roll image; (h) Hair catch image.

Moreover, to demonstrate the capability of the proposed image cipher in handling images with varying textures and shading, Fig 11 presents the original (plain) images. Additionally, Figs 12 and 13 display hte corresponding encrypted and decrypted images, respectively.

## 5 Performance and security analyses

This section employs diverse security validation metrics to objectively evaluate the effectiveness of the WCA-CMC technique proposed in this study. Furthermore, to ensure a comprehensive comparison, this study selects state-of-the-art works [58–61] for benchmarking the proposed approach against existing methodologies across multiple validation metrics.

### 5.1 Key space

Image ciphers having big and large key spaces ensure their safety from the brute-force attacks. In such attacks, hackers systematically attempt all possible keys of the cryptosystem until the correct one is identified. Cryptographic experts suggest that a key space of at least 2^100^ [62] is needed to withstand a potential brute-force attack. *q*_0_ = 0.4, η=0.2, r = 0.3, *x*_0_ = 0.5 and $\phi =2^{32}$ are part of the secret key used in the encryption algorithm. The computational precision of the system used in this study is 10^−14^. So, the total key space of the proposed WCA-CMC is $10^{14\times 5}$ × $2^{32}=2^{248}$. This value demonstrates strong resilience against brute-force attacks. Furthermore, Table 3 compares the key space of the proposed cipher with those of existing works in the field of image security. Unfortunately, our work couldn’t beat any of the chosen published works regarding the key space but we contend that we met the minimum threshold, i.e., $2^{248}\gg 2^{100}$.

**Table 3:** A key space comparison with other schemes.

Technique	Key space
Ours	$4.52\times 10^{74}\approx 2^{248}$
Ref. [58]	-
Ref. [61]	10^144^
Ref. [59]	2^430^
Ref. [60]	-

### 5.2 Statistical analysis

Statistical attacks are among the most commonly employed techniques by hackers to compromise ciphers. To demonstrate the resilience of the WCA-CMC against such attacks, this study employs two key analytical measures: histogram analysis and correlation analysis. Both the cartesian and polar histogram analysis will be carried out.

#### 5.2.1 Cartesian histogram

Images are composed of tiny pixels, each with a specific intensity value. A histogram systematically represents the number of pixels corresponding to each intensity level. The histograms of plaintext and cipher images exhibit distinct characteristics. In plaintext images, the histogram typically displays a curved distribution, whereas in cipher images, it appears uniformly smooth. This smoothness is a crucial security feature, as it enhances resistance against potential attacks. The smoother the histogram of a cipher image, the more secure it is against histogram-based attacks.

**Fig 14:**
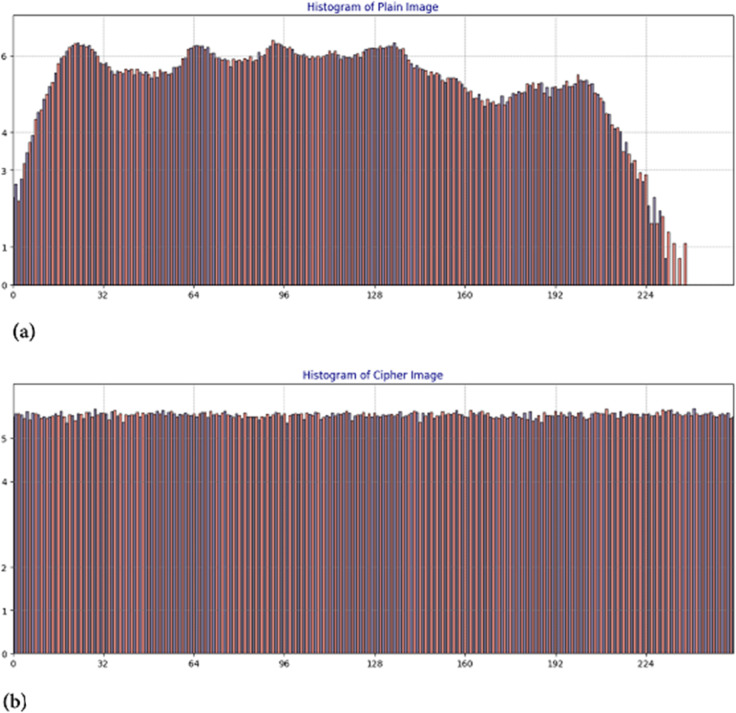
Cartesian histogram of Hailstones image: (a) Plaintext image; (b) Cipher image.

As observed in Fig 14, the histogram of the plaintext image has a curved distribution, while the cipher image exhibits a smooth, uniform histogram, demonstrating the robustness of the proposed cipher against histogram attacks.

Visual inspection of histograms alone is insufficient to accurately assess their curvature or smoothness. A more objective criterion is required for evaluation. Fortunately, variance has proven to be an effective metric for this purpose. Higher variance values indicate weaker security, while lower values correspond to stronger security [63].

Table 4 presents the histogram variance values for the cipher images of Hailstones, Flowers, Chair and Bride. The average variance across all selected images is 256.2446, while the variance for the Hailstones image is 251.9867. Both values are lower than 264.37 [64], demonstrating that the proposed encryption method offers superior security.

**Table 4:** Histogram variance results of cipher images.

Technique	Hailstones	Flowers	Chair	Bride	Average
Proposed	251.9867	261.0987	259.1910	252.7022	**256.2446**
Ref.[64]	264.37				

#### 5.2.2 Polar histogram analysis.

**Fig 15:**
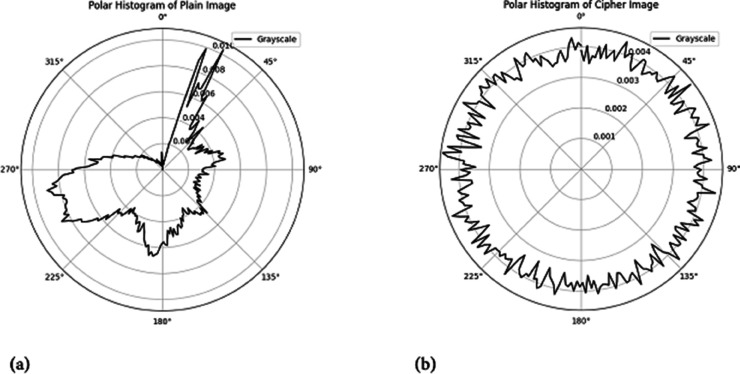
Polar histogram analysis of Hailstones image: (a) Plain image; (b) Cipher image.

The polar histogram of a plaintext image typically displays smooth variations with distinct peaks, indicating dominant intensity values associated with regions of consistent brightness or similar shading [65]. Conversely, the polar histogram of an encrypted image is expected to be more uniform, exhibiting a rougher texture and a more evenly distributed range of intensity values. This uniformity arises from the encryption process, which disrupts pixel intensity patterns to eliminate recognizable structures from the original image. Unlike the plaintext image, the encrypted image should lack prominent peaks, as encryption ensures an even redistribution of pixel values across the intensity spectrum. This randomness enhances security by preventing pattern recognition. Fig (15) highlights this distinction: the encrypted image (Fig 15b) demonstrates a uniform distribution without distinct peaks, whereas the plaintext image (Fig 15a) retains noticeable peaks. This contrast clearly illustrates the effectiveness of the proposed encryption method in concealing image features and strengthening security.

#### 5.2.3 Correlation coefficient analysis

Pixels in plaintext images exhibit a strong correlation with one another, which is the fundamental reason these images retain meaningful structure. However, when encryption is applied, the pixel intensities and positions undergo significant transformations, disrupting this inherent relationship. As a result, the strong connectivity between neighboring pixels is effectively dismantled.

To quantify the correlation between pixels, the following formula is utilized [66].

$$CC=\frac{N\sum_{j=1}^{N}(x_{j}\times y_{j})-\sum_{j=1}^{N}x_{j}\times \sum_{j=1}^{N}y_{j}}{\sqrt{\left(N\sum_{j=1}^{N}x_{j}^{2}-(\sum_{j=1}^{N}x_{j}{)}^{2})(N\sum_{j=1}^{N}y_{j}^{2}-(\sum_{j=1}^{N}y_{j}{)}^{2}\right)}}$$ (5)

**Fig 16:**
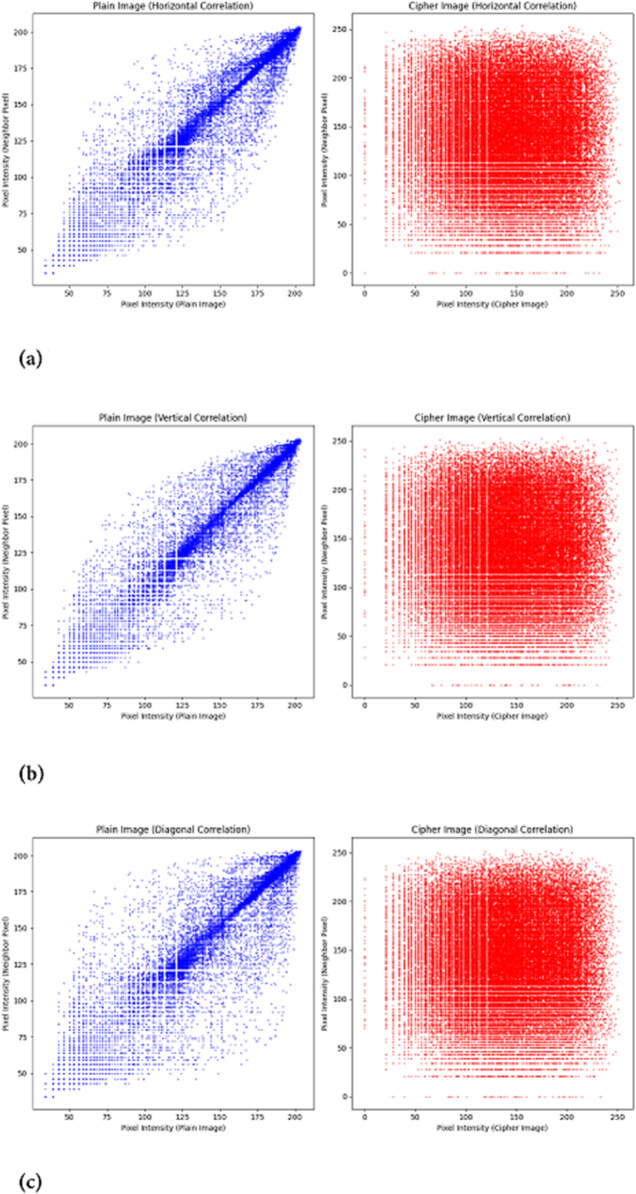
Correlation distribution of neighboring pixels in the specified direction for the Hailstones image: (a) Horizontal direction, plaintext image (left), cipher image (right); (b) Vertical direction, plaintext image (left), cipher image (right); (c) Diagonal direction, plaintext image (left), cipher image (right).

In this formula, the variable *N* indicates the pixels’ frequency in the given plaintext and cipher images. Apart from that, the variables *y* and *x* denote the intensity codes of those pixels. The correlation distribution of pixels in both cipher and plaintext is illustrated in Fig 16, considering three orientations: diagonal, horizontal, and vertical.

Table 5 presents the correlation coefficient between adjacent pixels for both plaintext and ciphered images of Hailstones. As observed, this value is nearly 1 for the plaintext image, indicating strong pixel correlation, while it approaches 0 for the ciphered image, confirming effective encryption. Additionally, Table 6 provides a comparative analysis, demonstrating that the proposed encryption method yields results on par with existing approaches.

**Table 5:** Correlation coefficient validation metric results.

Image	Plane	Direction		
		Horizontal	Vertical	Diagonal
Plain image of Hailstones	Red	0.9551	0.9263	0.9154
	Green	0.9503	0.9365	0.9276
	Blue	0.9423	0.9235	0.8721
Encrypted Hailstones image	Red	0.0036	0.0066	-0.0045
	Green	-0.0023	0.0054	0.0047
	Blue	-0.0038	0.0022	0.0049

**Table 6:** Comparative analysis of correlation coefficients across different encryption schemes.

Image	Technique	Direction		
		Horizontal	Vertical	Diagonal
Plain image of Hailstones		0.9492	0.9288	0.9050
Encrypted Hailstones image	Proposed	-0.0008	0.0047	0.0017
	Ref. [58]	0.0005	0.1313	-0.0047
	Ref. [61]	0.0013	-0.0009	-0.0023
	Ref. [59]	0.0033	0.0070	0.0027
	Ref. [60]	0.0002	0.0022	-0.0015

It is worth noting that around five thousand pairs of pixels were arbitrarily selected from the given cipher and plaintext images, and the correlation formula was applied to them. Ensuing results exhibit some variation due to the random nature of the selection process—certain pixel pairs may produce more favorable outcomes, while others may not.

### 5.3 Known plaintext, chosen plaintext,ciphertext only and JPEG attacks analyses

Cryptanalysts employ various techniques to compromise cryptosystems, with known plaintext, chosen plaintext, and ciphertext-only attacks being the most common [67]. Below, we outline the operational mechanisms of each attack. In a ciphertext-only attack, adversaries have access to only a limited number of ciphertexts. In contrast, a known plaintext attack provides them with pairs of corresponding plaintexts and ciphertexts. Chosen plaintext attack, however, grants attackers complete control over the encryption process, allowing them to generate as many ciphertexts as needed. If it can be demonstrated that the proposed image cryptosystem is resistant to a chosen plaintext attack, its resilience against ciphertext-only and known plaintext attacks follows naturally. This is because both of these attacks are essentially subsets of the chosen plaintext attack.

**Fig 17:**
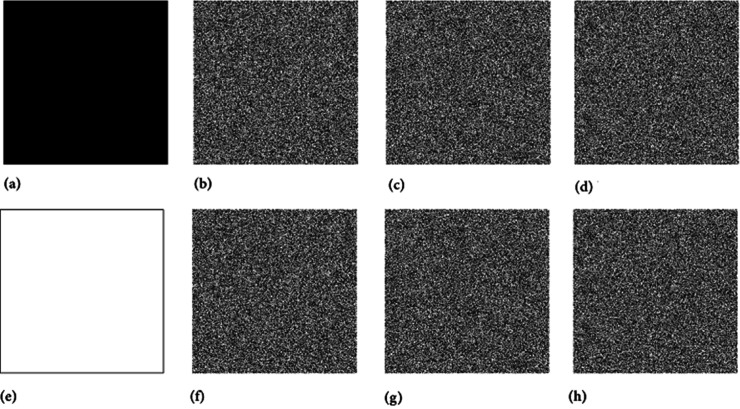
Chosen plaintext attack analysis through special images like Black and White: (a) Black plaintext image; (b) Black ciphertext image; (c) Ciphertext Hailstones image; (d) Retrieved Hailstones image with secret key from the Black image; (e) White plaintext image; (f) White ciphertext image; (g) Ciphertext Hailstones image; (h) Retrieved Hailstones image with secret key from White image.

To execute a chosen plaintext attack, adversaries may select a specific type of image, such as a Black image (Fig 17a), and encrypt it in an attempt to trace the secret key used in the encryption process. Once the Black image is encrypted (Fig 17b), the random numbers utilized in the encryption scheme are extracted. Following this, the attackers may choose any plaintext image of interest, such as the Hailstones image, and encrypt it (Fig 17c) by leveraging a known plaintext attack, using the key obtained from the encryption of the Black image. However, the attacker’s efforts would prove futile, as the cipher image of Hailstones cannot be decrypted using this key (Fig 17d). A similar experiment was conducted using a White image (Figs 17e to 17h), yielding the same results. These findings underscore the inherent robustness of the proposed image cryptosystem against chosen plaintext, known plaintext, and ciphertext-only attacks.

**Fig 18:**
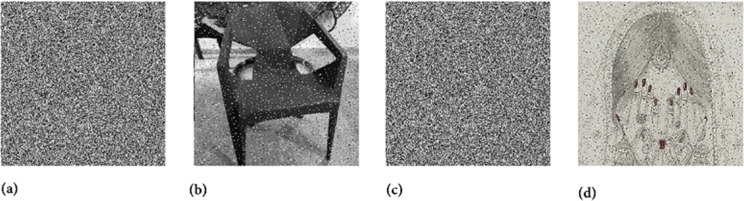
JPEG compression attack on cipher images: (a) Cipher image of Chair; (b) Decrypted image from (a); (c) Cipher image of Bride; (d) Decrypted image from (c).

Sometimes, a JPEG compression attack is employed by attackers against cipher images. In this attack scenario, the cipher image is first compressed in JPEG format. The compressed image is then decrypted using the proposed decryption algorithm. Fig 18 illustrates the robustness of the proposed image cipher under a JPEG compression attack. Although the reconstructed plain images appear slightly blurred, their content remains clearly identifiable. Table 7 presents the quantitative results for this important security metric. Our results are comparable with the existing works. [57,68,69].

**Table 7:** JPEG attacks analyses.

Work	Image	PSNR
Ref. [68]	Baboon	15.0854
Ref. [69]	Airplane	16.4162
Ref. [57]	Moon	14.5698
Proposed	Chair	15.7054
	Bride	15.2287

### 5.4 Information entropy analysis

Information entropy (IE), or simply Entropy, is a widely used metric to evaluate the effectiveness and resilience of cryptographic algorithms against potential attacks by hackers. In this analysis, entropy measures the degree of randomness or dispersion of pixel values within a given image. For a 256-level grayscale image, the maximum possible entropy value is 8. When the entropy of an encrypted image is very close to this ideal value of 8, it suggests that the encryption algorithm has effectively achieved both confusion and diffusion of the image’s pixels. This, in turn, indicates a high level of security, as the encrypted image exhibits a near-uniform distribution of pixel values, making it resistant to statistical attacks. The mathematical formulation of entropy, which underpins this concept, was first introduced in 1949 and is expressed as follows [70]:

$$H(m)=\sum_{i=0}^{2^{n}-1}p(m_{i})log\frac{1}{p(m_{i})}$$ (6)

Here, *H*(*m*) represents the information entropy of the signal *m*. Table 8 presents the entropy results for the selected images. The proposed algorithm demonstrates superior performance compared to the method described in [58] in terms of information entropy, indicating a higher level of randomness and security in the encrypted images.

**Table 8:** Information entropy results.

Scheme	Images	Plain	Cipher
Proposed	Hailstones	7.2507	7.9973
	Flowers	7.6942	7.9971
	Chair	7.2549	7.9973
	Bride	7.2104	7.9975
	**Average**	**7.3525**	**7.9973**
Ref. [58]	Baboon		7.9966
Ref. [61]	-		7.9999
Ref. [59]	Lena		7.99918
Ref. [60]	Lena		7.9992

### 5.5 Plaintext sensitivity (differential attack)

The attack under consideration is highly technical in both its approach and execution. In this assault, attackers obtain two versions (samples) of the plaintext image. One version remains unchanged, while in the other, a slight modification is made to the pixel intensity values. Both samples are then encrypted using the same encryption machinery. The intensity values of the pixels in these two encrypted samples exhibit a subtle relationship, which, upon further analysis, can potentially allow attackers to deduce the secret key. To counteract this type of attack, researchers have developed two key security metrics: *NPCR* (Number of Pixels Change Rate) and *UACI* (Unified Average Changing Intensity). These metrics quantify the effectiveness of an encryption algorithm in resisting such attacks. The mathematical formulations for NPCR and UACI are as follows:

$$NPCR=\frac{\sum_{t,u}J(t,u)}{M\times N}\times 100\%$$ (7)

The pair (*M*, *N*) of values refers to the size of the image we are dealing with. Furhter, *J*(*t*, *u*) is mathematically expressed as

$$J(t,u)=\left\{ \begin{array}{cc} 1, & \text{if}\;C(t,u)\neq C^{\prime }(t,u);\\ 0, & \text{if}\;C(t,u)=C^{\prime }(t,u). \end{array}\right.$$ (8)

$$UACI=\frac{1}{M\times N}\left[\sum_{t,u}\frac{\left|C(t,u)-C^{\prime }(t,u)\right|}{255}\right]\times 100\%$$ (9)

In the above equation, the variables *C* and $C^{\prime }$ represent the encrypted images without any changes in pixel values and with a slight modification in pixel values, respectively. Table 9 presents the experimental results of these metrics for the selected test images. The average values across all four test images are 99.6221% for NPCR and 33.6140% for UACI. These results are sufficiently close to the ideal values, indicating that the proposed image encryption algorithm possesses the necessary robustness to effectively resist potential differential attacks. Thus, we assert that the novel cipher is well-equipped to mitigate such security threats.

**Table 9:** Average NPCR and UACI values for different images.

Images	NPCR(%)	UACI(%)
Hailstones	99.6187	33.6065
Flowers	99.6211	33.6155
Chair	99.6298	33.6264
Bride	99.6190	33.6077
**Average**	**99.6221**	**33.6140**

Moreover, according to the Table 10, the proposed approach outperforms the works in [64,71–73] in terms of the NPCR metric and surpasses [64,71,72] with respect to the UACI metric.

**Table 10:** Comparison of NPCR and UACI metrics across different encryption schemes.

Scheme	Average NPCR(%)	Average UACI(%)
Proposed	99.6221	33.6140
Ref. [71]	99.6063	33.2985
Ref. [72]	99.6090	33.4727
Ref. [64]	99.6067	33.5000
Ref. [73]	99.6200	33.6900

### 5.6 Peak signal-to-noise ratio analysis

The Peak Signal-to-Noise Ratio (PSNR) quantitatively evaluates the extent of pixel value differences between the cipher and plain images. Its mathematical expression is given by:

{PSNR=20log10(255MSE)dBMSE=1m×n∑f=1m∑g=1n(Plain(f,g)−Cipher(f,g))2 (10)

In this formula, *m* and *n* represent the length and width of the given images, respectively. Additionally, *Plain*(*f*, *g*) and *Cipher*(*f*, *g*) denote the intensity values of the pixels in the plain and cipher images at coordinates (*f*, *g*). The term *MSE* refers to the mean squared error, where a higher *MSE* value enhances security effects. Conversely, a lower *PSNR* value is desirable, as these two parameters are inversely related.

Table 11 presents the *PSNR* results of the proposed method alongside various studies from the literature. According to the table, the *PSNR* value approaches infinity (∞) when the formula is applied to plain and decrypted images. This indicates that the plain and decrypted images are identical due to *MSE* = 0, signifying no distortion between the restored and original images. In the table, “O-C” denotes the original and cryptic images, while “O-D” refers to the original and decrypted images.

**Table 11:** Peak signal to noise ratio results and comparison.

		Hailstones	Flowers	Chair	Bride	Average
Ours	PSNR (O-D)	∞	∞	∞	∞	∞
	PSNR (O-C)	7.1542	9.2911	8.9961	8.8342	**8.5689**
Ref. [74]	PSNR (O-C)	8.62725				
Ref. [75]	PSNR (O-C)	8.6285				

Furthermore, the *PSNR* results for the Hailstones image using the proposed approach WCA-CMC outperform those of other studies [74,75]. Therefore, we can conclude that the proposed method demonstrates superior performance compared to existing approaches.

### 5.7 Mean absolute error (MAE)

MAE is another crucial security validation metric for evaluating the effectiveness of image cryptosystems. When a plaintext image undergoes encryption, its pixel values and positions are significantly altered. This parameter quantitatively measures the extent of these transformations. To assess this, both the cipher and plaintext images are used as inputs. The corresponding equation is given below.

$$MAE=\frac{1}{m\times n}\sum_{x=1}^{m}\sum_{y=1}^{n}abs(Cipher(x,y)-Plain(x,y))$$ (11)

In this formula, *Plain* and *Cipher* represent the plaintext and cipher images, respectively, with (*m*, *n*) denoting their dimensions. A higher value of this security parameter indicates stronger encryption effectiveness.

Table 12 presents the results obtained using the proposed scheme, which outperform those reported in [75].

**Table 12:** Mean absolute error findings.

Image	MAE
Hailstones	89.4347
Flowers	82.0984
Chair	100.2123
Bride	91.9255
**Average for all images**	**90.9177**
Ref. [75]	77.4998

### 5.8 Noise and data loss threats

Once cipher images are produced, they are typically transmitted through such channels which may be vulnerable, hence making them susceptible to various attacks, such as noise and data cropping.

**Fig 19:**
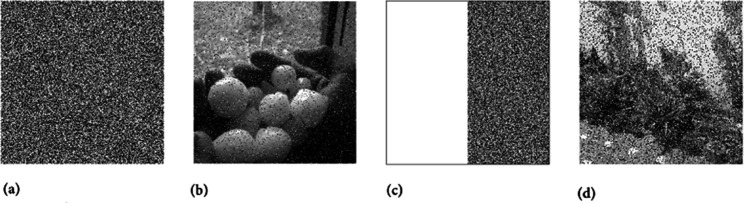
Noise and data crop attacks analysis: (a) Cipher Hailstones image (Noise density 0.1 added); (b) Decrypted image from (a); (c) Cipher Flowers image with data loss by $\frac{1}{2}$; (d) Decrypted image from (c).

In a noise attack, random noise is introduced into the cipher images, altering pixel intensity values. As a result, when the original plain images are reconstructed at the destination, they may not perfectly match the originals due to pixel distortion. For evaluating the resilience of WCA-CMC against noise attacks, artificial noise with different intensities was added, as illustrated in Fig 19. Specifically, noise intensity of 0.1 was applied to the cipher image of Hailstones. Despite the noise, Fig 19b shows that the decrypted image remains recognizable, demonstrating the robustness of the proposed image cipher against noise attacks.

In a cropping attack, as its title indicates, some percentage of the cipher image is lost during transmission from one point to the other. To simulate this scenario, a substantial portion of pixel data was deliberately removed from the cipher image (Fig 19c). After applying the decryption algorithm, the restored image (Fig 19d) remains identifiable, indicating that the WCA-CMC is resilient against cropping attacks.

These findings confirm that the WCA-CMC is robust enough to withstand both noise and cropping attacks effectively.

### 5.9 Computational time analysis

One of the key contributions of this study is that the proposed novel image cipher operates significantly faster than many existing methods in the literature. The implementation was carried out using Python 3 tool on a Windows operating system. The system specifications include an Intel(R) Core(TM) i5-4210U CPU running at 1.70 GHz (boosting up to 2.40 GHz) with 8 GB of installed memory.

As shown in Table 13, the encryption of the Hailstones image takes just 0.8051 seconds, while the average encryption time for all tested images is 0.8053 seconds. Furthermore, a comparison with existing studies confirms that the proposed method outperforms the works in [71,76] in terms of computational efficiency.

**Table 13:** Encryption speed of the proposed technique and its comparative analysis with existing methods.

Technique	Image	Speed in seconds
Ours	Hailstones	0.8051
	Flowers	0.8267
	Chair	0.8823
	Bride	0.7072
	**Average**	**0.8053**
Ref. [71]	Lena	2.5607
Ref. [76]	Lena	3.1143
Ref. [73]	-	0.067230

## 6 Discussion

The experimental results strongly support the research hypothesis that combining Wireworld Cellular Automaton (WCA) with one-dimensional logistic and piecewise linear chaotic maps significantly enhances the security of image encryption. The chaotic maps effectively generate high-quality, key-dependent pseudo-random sequences that introduce substantial unpredictability into the encryption process. These sequences initialize and influence the evolution of the Wireworld automaton, whose localized interactions contribute to effective pixel-level scrambling and dynamic diffusion.

The observed entropy value of 7.9975 is close to the ideal value of 8, indicating excellent randomness in the encrypted images. Additionally, the histogram variance of 251.9867 confirms uniform pixel value distribution, further evidencing the effectiveness of the scrambling process. Correlation coefficients between adjacent pixels are substantially reduced, and statistical analyses—including Cartesian and polar histogram flattening—demonstrate the cipher’s resistance to statistical attacks. Furthermore, the encryption shows strong resilience against differential attacks, with NPCR and UACI values within optimal ranges.

Collectively, these findings confirm that the proposed WCA-CMC algorithm not only upholds the core hypothesis but also offers competitive security metrics when compared to conventional schemes. The integration of CA-based structure with chaos-driven randomness presents a viable, lightweight, and practical approach for real-world image encryption applications.

## 7 Conclusion

In this study, we have proposed a novel image encryption algorithm that integrates the Wireworld cellular automaton, the 1D logistic chaotic map, and the piecewise linear chaotic map to enhance security and resilience against cryptographic attacks. The synergistic combination of these techniques effectively ensures both confusion and diffusion, making the encryption process highly robust. The comprehensive security analyses, including key space evaluation, correlation coefficient analysis, entropy measurement, and PSNR assessment, confirm the effectiveness of the proposed cipher in protecting digital assets from various attacks. Experimental results demonstrate that the algorithm achieves strong security characteristics while maintaining computational efficiency, making it a practical solution for real-world applications. Its applicability extends to critical domains such as healthcare, military communications, and multimedia security, where image confidentiality is paramount. Given its strong resistance to cryptographic attacks and promising performance, this approach represents a significant advancement in the field of image encryption. Future research may focus on optimizing the algorithm’s computational complexity, extending it to other forms of multimedia encryption, and exploring its implementation in resource-constrained environments such as IoT devices and edge computing. The findings of this study contribute to the ongoing efforts in developing secure and efficient cryptographic solutions for the digital age.
